# Sex Differences in Stress Response: Classical Mechanisms and Beyond

**DOI:** 10.2174/1570159X22666231005090134

**Published:** 2023-10-05

**Authors:** Georgia E. Hodes, Debra Bangasser, Ioannis Sotiropoulos, Nikolaos Kokras, Christina Dalla

**Affiliations:** 1 Virginia Tech, School of Neuroscience, Blacksburg, VA, USA;; 2 Center for Behavioral Neuroscience, Georgia State University, Atlanta, GA, USA;; 3 Institute of Biosciences & Applications NCSR “Demokritos”, Athens, Greece;; 4 Life and Health Sciences Research Institute (ICVS), School of Medicine, University of Minho, Campus de Gualtar, 4710-057 Braga, Portugal;; 5 Department of Pharmacology, Medical School, National and Kapodistrian University of Athens, Athens, Greece;; 6 First Department of Psychiatry, Eginition Hospital, Medical School, National and Kapodistrian University of Athens, Athens, Greece

**Keywords:** Stress, sex differences, immune, neurodegeneration, antidepressants, HPA axis, glucocorticoids

## Abstract

Neuropsychiatric disorders, which are associated with stress hormone dysregulation, occur at different rates in men and women. Moreover, nowadays, preclinical and clinical evidence demonstrates that sex and gender can lead to differences in stress responses that predispose males and females to different expressions of similar pathologies. In this curated review, we focus on what is known about sex differences in classic mechanisms of stress response, such as glucocorticoid hormones and corticotrophin-releasing factor (CRF), which are components of the hypothalamic-pituitary-adrenal (HPA) axis. Then, we present sex differences in neurotransmitter levels, such as serotonin, dopamine, glutamate and GABA, as well as indices of neurodegeneration, such as amyloid β and Tau. Gonadal hormone effects, such as estrogens and testosterone, are also discussed throughout the review. We also review in detail preclinical data investigating sex differences caused by recently-recognized regulators of stress and disease, such as the immune system, genetic and epigenetic mechanisms, as well neurosteroids. Finally, we discuss how understanding sex differences in stress responses, as well as in pharmacology, can be leveraged into novel, more efficacious therapeutics for all. Based on the supporting evidence, it is obvious that incorporating sex as a biological variable into preclinical research is imperative for the understanding and treatment of stress-related neuropsychiatric disorders, such as depression, anxiety and Alzheimer’s disease.

## 1 INTRODUCTION

Stressful experiences are a part of life, but the way people respond to stress varies. In general, the stress response itself is meant to be adaptive, particularly to acute stressors, as it allows for mobilizing energy stores while suppressing growth reproduction and drives the immune system to prepare the body to deal with a threat environment [1, 2]. In some cases, prior stress can promote resilience to future stressors, a phenomenon called stress inoculation [3, 4]. However, the effects of a maladaptive response to stress can be multidimensional and may include different neurobiological and psychological outcomes, like alexithymia [5]. For example, maladaptive responses have recently been observed in stressed healthcare professionals during the recent COVID-19 pandemic [6]. Moreover, chronic stress or an unchecked stress response is a risk factor for a range of psychiatric and neurodegenerative disorders, including major depression and Alzheimer’s disease (AD) [7-9].

There are many factors that can contribute to variability in how people respond to stress, ranging from genetic factors to environmental (*e.g*., social support). Here, we focus on how sex/gender can lead to differences in stress responses that predispose males and females to different pathology.

Evidence that sex/gender can influence stress reactions comes, in part, from epidemiological data that reveal that disorders linked to stress hormone dysregulation occur at different rates in men and women [10]. For example, rates of major depression are nearly twice as high in women as in men [11, 12]. Women are also more likely to suffer from anxiety disorders than men, with a lifetime female-to-male prevalence ratio of 1.7:1 [13]. This sex/gender disparity is observed in various disorders linked to high levels of perceived stress and trauma and stress hormones, including generalized anxiety disorder, panic disorder, social anxiety disorder, specific phobias, and post-traumatic stress disorder (PTSD) [14, 15]. Other neurological and medical conditions - such as AD, migraines, insomnia, and irritable bowel syndrome - that are more common in women than men are often comorbid with depression and anxiety, perhaps suggesting some common underlying pathology [16-25]. However, it is an oversimplification to assume that women are simply more vulnerable to stress. There are disorders exacerbated by stress, such as schizophrenia, that have a male bias in rates and age of onset [26-28]. Additionally, psychological and sociocultural factors play a role. For example, diagnostic criteria, which for psychiatric disorders are completely symptom-based can influence outcomes. One study using a broader diagnosis for depression that includes additional symptoms, such as anger attacks/aggression, substance abuse, and risk-taking, found that this expanded criterion eliminates sex disparities in disease prevalence [29].

Based on epidemiological data alone, it is difficult to obtain a clear picture of whether sex-related variables, such as gonadal hormones, are important to consider in the understanding of the etiology of stress-related disorders and treatment development. To address these limitations, the field has turned to non-human animal models of stressor exposure to determine whether there are sex differences in stress responses relevant to human health. These models are crucial in driving drug development [30], so it may be surprising that, historically, they excluded female animals [31]. However, in response to pressure from funding agencies in the United States of America and Canada [32], in the past five years or so, more basic and preclinical studies have included both sexes in their designs, although there are still major gaps in proper sex comparisons [33, 34], as well as targeted funding [35].

Despite the limited data including female subjects, here we focus on what is known about sex differences in classic mechanisms of responding to stress. We also talk about more recently recognized regulators of stress and disease, such as the immune system, epigenetic mechanisms, neurodegeneration and sex differences therein. Finally, we discuss how understanding sex differences in stress responses can be leveraged into novel therapeutics that better treat psychiatric and neurological disorders in everyone.

## 2 CLASSICAL MECHANISMS AND STRESS RESPONSES

The hypothalamus-pituitary-adrenal (HPA) axis is activated, in part, to provide energy to the body in response to stress, and its dysregulation has been implicated in stress-related disorders. Sex differences in the HPA axis have been described in detail [36], with female rodents having higher plasma corticosterone, the most abundant glucocorticoid found in rats, in comparison to males [37-39]. However, this sex difference seems to depend on several factors, such as strain, age, time of sampling, housing conditions, diet and estrous cycle phase, reproductive status, *etc*. [39-42]. Interestingly, globulin corticosteroid binding, which determines the amount of the unbound, active corticosterone that reaches the brain, is also sex-differentiated [43] and is influenced by stress [44].

Stress-induced activation of the HPA axis is more robust in female rats than in males, but this activation does not seem to correlate with the female behavioral response to stress [36, 45, 46]. For example, stress enhances corticotropin-releasing factor (CRF), ACTH, and corticosterone more in female than male rats [41, 47, 48], but when surgical adrenalectomy is performed in female rats, and corticosterone is substituted, resulting in stable corticosterone levels, females continue to demonstrate stress associated behavior in the FST. This finding suggests that generally, the female behavioral response to stress, as evidenced in this case from the immobility, swimming and climbing FST behaviors, is less influenced by the HPA axis, whereas in males, their behavioral response to stress more accurately tracks the HPA axis (dys)function [45].

Similarly, in another study, adrenalectomy also did not alter a well-described stress effect in female rats. Specifically, associative learning in trace eyeblink condition is decreased in female rats as a consequence of acute stress exposure (30 min of tail shock in a restrainer tube) that has been applied 24 hours in advance. However, in male rats, the same surgery, which prevents HPA axis activation, abolished the effects of acute stress in enhancing male eyeblink conditioning [49]. Notably, these acute stress effects require the hippocampus in both sexes [50] and cause respective sex-differentiated stress responses in the density of spines in the CA1 area of the hippocampus, *i.e*., decrease in females and increase in males. The phase of the estrous cycle is important in determining the female stress effect on learning and spine density on the hippocampus, which is a measure of synaptic plasticity. Specifically, this is evident when females are stressed in the proestrous phase of the cycle and are sacrificed or begin testing in the diestrous phase when estrogens are low [51]. Interestingly, this female stress effect is also dependent on the organizational effects of gonadal hormones, as the female response can be masculinized with one injection of testosterone on the first day of birth [52]. Both associative learning and spine density, in response to acute stress in adult masculinized female rats (which do not have a cycle) are enhanced in a similar fashion to male rats [53, 54].

Sex differences in neurotransmitter levels are also present in response to stress [40, 55, 56]. In particular, rats exposed to the FST, which consists of two sessions of swim stress on two consecutive days, had enhanced serotonergic activity. This was indicated by an increased 5-HIAA/5-HT turnover ratio in the hippocampus and levels of serotonin’s metabolite 5-HIAA in the prefrontal cortex on the second day [39]. Interestingly, serotonergic activity in the prefrontal cortex correlates with behavior in the FST, whereas hippocampal serotonin does not [57]. In response to 6 weeks of chronic mild stress, only female rats exhibit decreased serotonergic activity in the hippocampus and the hypothalamus, which might be linked with a higher rate of female depression and stronger response to SSRIs [40].

Sex differences in the response of the dopaminergic system to short-term stress also occur. Specifically, dopaminergic activity is enhanced in females following the forced swim and this has been considered as an adaptive mechanism [39, 58]. Previous studies have also shown that stress exposure influences amino acid levels in the prefrontal cortex and the hippocampus of rats, two brain regions involved in stress-related and affective disorders [59-61]. In particular, exposure to two sessions of swim stress enhanced glutamate, glutamine, GABA and taurine levels in female rats. In males only GABA and taurine levels were enhanced [39]. It has been suggested that stress-induced glutamatergic and GABAergic enhancements are linked with changes in serotonergic and dopaminergic activity in the PFC [62, 63].

Overall, enhancements in neurotransmitter levels in response to short-term or acute stress can be considered as a typical adaptive response, whereas chronic stressful experience results in decreased neurotransmission that contributes to the neurobiology of stress-related and affective disorders.

## 3 SEX DIFFERENCES IN THE CORTICOTROPIN RELEASING FACTOR - CRF SYSTEM

CRF dysregulation is implicated in psychiatric disorders, such as major depressive disorder (MDD), and neurodegenerative disorders, such as AD. In stress-related psychiatric disorders, high CRF levels are found in the cerebrospinal fluid (CSF) of humans with depression and PTSD [64, 65]. In MDD, the elevated CRF in CSF normalizes with successful treatment, correlating CRF levels with symptomatology [66]. In postmortem brain tissue from people with depression, high levels of CRF are found in the paraventricular: nucleus (PVN) and in neuromodulatory regions, including the raphe and locus coeruleus (LC) [67, 68]. CRF is also linked to neurodegenerative disorders. In AD, chronic stress, which leads to high CRF levels, increases the risk for the disorder [69, 70]. However, CRF immunoreactivity is reduced in postmortem tissue from people with AD, but this is accompanied by CRF receptor upregulation, perhaps as a compensatory response to counter the lower CRF levels [71, 72]. The impact of these changes is unclear, but it has led to the theory that initial CRF hypersecretion due to stress increases AD risk, but the lasting dysregulation of central CRF may decrease its tone on important regions for memory and cognition, contributing to cognitive deficits [69]. As noted, MDD and AD occur more often in women, but unfortunately, sex/gender differences in CRF in patients with these conditions have not been investigated. In neurotypical populations, peripheral administration of CRF causes an increased ACTH response in women compared to men [73]. This could indicate that women have a greater HPA axis response to CRF release, which could bias them towards these disorders linked to CRF.

Despite the paucity of data on sex differences in CRF in humans, there are many rodent studies showing that, in regions relevant to affect and cognition, there are sex differences in CRF that range from the inputs that regulate CRF neurons to CRF’s postsynaptic efficacy [74]. There is some evidence that female rats have greater CRF expression than males in the PVN [48, 75]. In target regions of the PVN, including the pituitary and the medial septum, CRF binding protein (CRF-BP), which binds free CRF to reduce its bioavailability, is higher in female than male rodents [76, 77]. This increase in female CRF-BP may help compensate for higher levels of CRF released into these regions from the PVN.

There are two types of CRF receptors: CRF_1_ and CRF_2._ Global knockout studies have found largely opposing effects of these receptors where CRF_1_ initiates the HPA axis and increases anxiety, while CRF_2_ mediates the duration of the HPA axis response, promoting stress recovery and reducing anxiety [78-80]. There are sex differences in CRF receptor function. For example, CRF_1_ binding typically reflects receptor number, and it is increased in the cortex, nucleus accumbens, and amygdala of adult female rats [81]. In the rostral portion of the anteroventral periventricular nucleus of the hypothalamus, a region that regulates maternal behavior, female mice have more CRF_1_-positive neurons than males, and these sex differences in exacerbated by chronic variable stress [82, 83]. There are also sex differences in CRF_2_ binding, which tends to be male-biased in that higher levels of CRF_2_ binding in the bed nucleus of the stria terminalis, amygdala, and hypothalamus are found in male compared to female rats [81]. Given the differential roles of CRF_1_ and CRF_2_, these sex differences in binding may bias females toward stress reactivity and anxiety and males towards stress recovery. In addition to differences in receptor amount, sex differences in the distribution of CRF receptors on different cell types have also been reported. In the dorsal raphe, CRF_1_ receptors have a higher colocalization with parvalbumin-containing GABA neurons in male mice, while in the hippocampal CA1 region, there is great CRF receptor colocalization with delta opioid receptor-containing dendrites in female rats [84, 85]. Sex differences in receptor distribution can influence how regions respond to stress and their downstream effects on efferent targets.

CRF receptors are G-protein coupled receptors, and while they preferentially bind the Gs protein and signal through the cyclic AMP (cAMP) protein kinase A (PKA) signaling pathway, they can also bind other G proteins and β-arrestin [86, 87]. Thus, the downstream effects of CRF receptors are not only regulated by their receptor number and localization but also by their signaling. Sex differences in CRF_1_ signaling are found in the LC, a noradrenergic-containing nucleus that projects to many regions, including the cortex, to increase levels of arousal [88-90]. Specifically, CRF_1_ receptors in the LC signal more through the cAMP-PKA pathway in females compared to male rats, which increases the sensitivity of female LC neurons to CRF [91]. This sex difference in sensitivity is linked to sex differences in cortical network activity, as CRF in the LC increases theta oscillations in the medial prefrontal cortex and its coherence with the orbitofrontal cortex in females but not in males [92]. Under acute or moderate stress, this increased sensitivity of LC neurons to CRF in females may help promote alertness and cognitive processing. However, under conditions of CRF hypersecretion, it could lead to hyperarousal, a negative state of being on edge that contributes to some symptoms of depression and PTSD and may be more prominent in women [93-96].

**Fig. (1):**
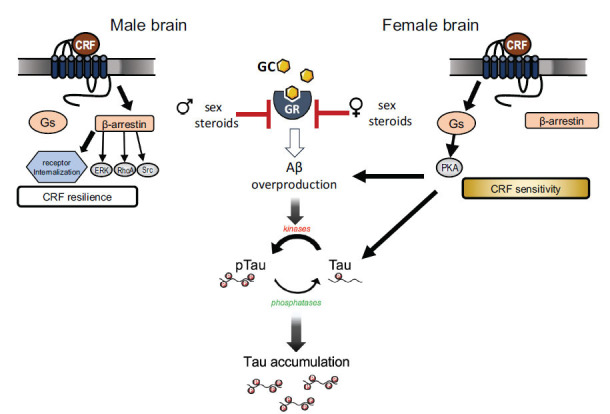
CRF and glucocorticoid signaling interplay in male and female AD brain. A schematic representation of the complex interplay between sex hormones, corticotrophin-releasing factor (CRF), and glucocorticoid (GC) signaling in male and female Altzheimer disease (AD) brain. Under chronic stress conditions, both CRF and GC receptor (GR) signaling seems to participate in the stress-driven Aβ overproduction as well as Tau hyperphosphorylation and accumulation in the AD brain with female brain exhibiting a CRF-Gs-PKA cascade activation that contributes to both AD pathomechanisms. Note the counteracting role of sex steroids in stress-induced GR activation and downstream induction of AD-related pathomechanisms, suggesting that reduction of male or female sex steroids (*e.g*., by aging) and the concomitant exposure to prolonged stress and high GC levels increase Aβ overproduction and/or Tau hyperphosphorylation and accumulation thus, endangering neuronal function and triggering AD neuropathology.

Similar sex differences in CRF_1_ are also found in the cortex, where CRF_1_ receptors are more highly coupled to Gs in females but to β-arrestin in males [91]. β-arrestin can activate its own suite of signaling cascades that are often distinct from those activated by G-proteins [97, 98]. Using a phosphoproteomic approach in CRF overexpressing (CRF-OE) mice, it was found that CRF hypersecretion increased activation of phosphopeptides in cortical Gs signaling pathways in females and β-arrestin signaling pathways in males [99]. This indicates that the signaling of the CRF_1_ is sex-biased [100]. An additional discovery was that CRF-OE female mice had an overrepresentation of phosphopeptides in the AD pathway and increased tau phosphorylation in the cortex [99]. In a mouse model of AD pathology, female mice that express human APP and also have an overexpression of CRF in the forebrain have an increased formation of amyloid β plaques and cognitive impairments relative to males [99]. Together, these studies suggest that CRF hypersecretion can bias females toward AD pathology (Fig. **1**).

Given the link between high levels of CRF and brain disorders, there has been an effort to develop CRF_1_ receptor antagonists to treat psychiatric disorders [101]. These antagonists were initially tested in preclinical studies using male rodents [102-106]. At the time, there was no evidence that the CRF_1_ receptor could signal differently in males and females because researchers were not using female rodents in their studies. However, this different signaling likely reflects a sex difference in the conformation of the CRF_1_ receptor, which could alter the efficacy of antagonists. The one clinical trial that showed some efficacy of CRF_1_ antagonists in depression included only men [107]. The other unsuccessful CRF_1_ antagonist clinical trials for depression tested CRF_1_ antagonists in mixed-sex/gender groups or only in women [36]. Unfortunately, the data from the mixed-sex/gender trials was not disambiguated by sex, so we are unaware whether these drugs were actually effective in men in these other trials. These findings highlight the problems with excluding females from basic and preclinical research and not disambiguating data by sex/gender in clinical trials. Moreover, they underscore that sex differences in pharmacodynamics, such as receptor function, should be assessed and considered in developing therapeutics.

## 4 THE PRECIPITATING ROLE OF CHRONIC STRESS ON NEURODEGENERATION: THE ROLE OF SEX

As noted, clinical studies have suggested that lifetime stress is associated with the early onset of AD pathology [108, 109]. In addition to CRF, other stress hormones, such as glucocorticoids (GCs), are associated with the initiation and progression of AD. For instance, chronic stress may advance the age of onset of the familial form of AD, while cortisol levels in AD patients correlate with their memory deficits [110-112]. In addition, high cortisol levels, the abundant GC in humans, are commonly found in AD patients’ plasma, saliva, and CSF [113-115], while AD patients also show higher total daily secretion of cortisol [116]. It is noteworthy that female AD patients show higher cortisol levels than male patients [117], suggesting that a sex difference in the stress response may contribute to the increased risk of women for AD with a potential role for both GC and centrally active CRF.

Focusing on the accumulation of amyloid β (Aβ), a molecular hallmark of AD brain, a recent clinical study that used Pittsburgh compound B positron emission tomography (PiB - PET) technology correlated high cortisol levels with elevated Αβ levels in the AD brain [118]. In line with clinical evidence, animal studies showed that elevated GC levels or exposure to chronic stressful conditions increased the levels and accumulation of Aβ in the brain, resulting in impaired cognitive function [119, 120]. In addition, neuronal mechanisms involved in the degradation or excretion of Aβ may be inactivated by stress; *e.g*., chronic stress affects the brain's excretion properties by reducing the expression of aquaporin 4 (AQP4) protein, exacerbating the accumulation of Aβ [121]. As mentioned above, CRF may also play a critical role in the stress-driven precipitation of AD, with females being more vulnerable to males [99, 122]. However, the role of GC and their signaling in the interplay of stress and sex in AD precipitation remains mainly unclear.

Different studies suggest that chronic stress also triggers different parameters of Tau pathology, the other hallmark of the AD brain. Exposure of animals to chronic stressful conditions resulted in the hyperphosphorylation of Tau and its accumulation in both neuronal dendrites and synapses, leading to neuronal malfunction and impairments of synaptic signaling [123-125]. This stress- or GC-driven accumulation of abnormal forms of Tau protein may occur *via* the inhibition of different degradation mechanisms of Tau. For instance, the autophagy mechanism and the endolysosomal degradation pathway are inhibited under stressful conditions, and this leads to pathological accumulation of Tau protein and neuronal malfunction in the brain of experimental AD rodent models [126, 127]. In addition, molecular chaperones, *e.g*., heat shock proteins (Hsp90 and Hsp70) involved in Tau degradation, are also shown to be dysregulated by chronic stress [124]. Hsp90 and Hsp70 maintain GC receptors in a high-affinity state, thus suggesting a point at which GC/GC receptor signaling and Tau degradation machinery can intersect. Interestingly, the above mechanism related to Tau accumulation is suggested to be involved in the increased vulnerability of the female hippocampus to the detrimental effect of chronic stress. Compared to males, females exhibited higher levels of Tau pathology and neuronal malfunction, as well as cognitive impairment in response to prolonged stress with particular role for molecular chaperones [124]. Note that prolonged exogenous administration of GCs presents similar effects, demonstrating their central role in the pathological process triggered by chronic stress [125, 128]. It is now clear that the HPA axis, GCs, and CRF are involved in the regulation of AD pathological mechanisms under exposure to prolonged chronic stress, resulting in the accumulation of Aβ and Tau protein in the brain [129]. For example, animal studies suggest the involvement of CRF receptors in stress-induced hyperphosphorylation of Tau. As noted, CRF overexpression increases the hyperphosphorylation of Tau with a greater effect in females than males [130, 131].

Notably, it is important to mention here a potential interaction of sex hormones with different components of stress (*e.g*., GC and GC signaling). For instance, it is suggested that loss of the neuroprotective effect of estrogens could contribute to the increased vulnerability of females to stress-driven AD brain pathology [132], as de-masculinization of neonatal male AD Tg mice narrows the gender gap in terms of Aβ pathology [133]. Moreover, there is strong evidence for an interplay between GC and sex steroids, in particular with respect to the regulation of neuroendocrine function and behavior. Previous studies demonstrate that the depletion of male gonadal steroids exacerbates the GC-driven Tau hyperphosphorylation [134], while clinical evidence recently showed that testosterone can counteract GC-induced hippocampal atrophy and memory deficits in middle-aged men [135]. Given that age is a risk for AD and that GC and sex steroid levels are inversely regulated (increased and decreased, respectively) during aging [136], future studies should further clarify the molecular underpinning of the complex interplay between sex, aging and stress in the precipitation of AD, as well as further dissect the GC and CRF contribution to the stress-driven AD brain pathology.

## 5 BEYOND CLASSICAL STRESS RESPONSES: SEX DIFFERENCES IN GENETICS, EPIGENETICS, AND IMMUNE RESPONSE TO STRESS

Translational studies have identified genetic, epigenetic, and immune mechanisms that contribute to stress susceptibility and are relevant to human mood disorders. Preclinical studies often use stress to induce behavior that overlaps with symptoms/domains of depression. These stress paradigms vary with different laboratories using forms of social, variable, or unpredictable stress applied chronically to induce changes in behavior [137]. In some of these paradigms, not only do males and females engage in different behaviors in response to stress, but the underlying transcriptional response is different or even opposite [138-144]. Even when depression-like behaviors are similarly induced in male and female mice, there is less than 30% overlap in stress-induced gene expression in the nucleus accumbens (NAc) and pPFC [145]. In humans’ different transcriptional signatures have been identified in these brain regions of men and women with depression [146, 147]. A variety of epigenetic mechanisms contribute to transcriptional sex differences in both humans with depression and rodents exposed to stress. These include sex-specific regulation by DNA methyltransferase (DNMTs), histone modifications, microRNAs (miRNA) and long non-coding RNA (lncRNA).

DNMTs are enzymes that covalently link a methyl group to the 5’ position of cytosine nucleotides of DNA, resulting in the suppression of gene expression [148]. There are several classes of DNMTs, including DNMT1, which maintains methylation between progenitor and daughter cells [149]. DNMTs 3a & b are involved with *de novo* methylation, for which they methylate sites that were previously unmethylated and/or recruit methylation binding domain proteins to produce a variety of histone modifications [149]. DNA methylation is a component of typical development, and constitutive knockout is lethal. Rodent studies suggest males and females have different baseline patterns of methylation, which is important to feminizing the brain and its response to a variety of stimuli [140, 150]. The NAc, a key region involved in reward, DNMT3a over-expression shifts both sexes to be more sensitive to stress [140]. In males, DNMT3a over-expression, in the absence of stress, increases spine density in the NAc, similar to the effects of either cocaine administration or social stress [151, 152]. Blocking DNMT3a activation by 6-day variable stress in the NAc of female mice shifts their behavior to a male-like response and promotes behavioral resilience [140]. Bulk sequencing of the NAc demonstrated that this manipulation removed many of the pre-existing transcriptional differences induced by stress between males and females, resulting in greater overlap of transcription. When intracerebral DNMT3a was repressed in female mice during early prenatal development, they engaged in male-like sex behaviors in adulthood after priming with testosterone and exposure to a receptive female [150].

While DNMT3a knockdown promoted a male-like response to stress in females, stress can be blocked in males by decreasing DNMT1 expression and increasing histone modifications [153]. Male mice given the phytochemicals dihydrocaffeic acid (DHCA) and malvidin-3′-O-glucoside (Mal-gluc) are resilient to social defeat stress and have reduced interleukin-6 (IL-6) expression in the periphery and decreased spine density in the NAc. In both male and female mice exposed to social defeat stress or variable stress, DHCA/Mal-gluc blocks the effects of stress on behavior, but through different mechanisms resulting in different transcriptional changes and alterations of different peripheral cytokines [153, 154].

Histone modifications result from additions or removal of marks on the N-terminal tails of the histone core in nucleosomes [155-158]. These modifications can open or close chromatin structures, resulting in the ability to express or suppress gene transcription [157, 159, 160]. Histone modifications during development have long-lasting effects by sex-specifically shaping brain regions and their subsequent stress response. The bed nucleus of the stria terminalis (BNST) is a sexually dimorphic structure involved in emotional responses to stress. It is masculinized through histone acylation in combination with testosterone during an early postnatal critical window [161]. Neurons in the female BNST undergo apoptosis during this critical window, resulting in a smaller volume in females compared to males [161]. If male mice are treated during this time point with a histone deacetylase inhibitor (HDAC), they have a feminized BNST [161]. Injection of testosterone into females during this same time will produce a male-like BNST [162]. In adulthood, masculinized females respond to acute stress like males, resulting in enhanced learning ability [162, 163], suggesting that epigenetic modification of the BNST may be involved in stress resilience. Histone modifications in adulthood can alter the behavioral response of male rodents to social stress through hyperacetylation of the BDNF promoter in the hippocampus, which also occurs following chronic antidepressant treatment [164]. Sex differences in long-term retrieval of fear memory are also dependent on histone modifications, specifically acetylation of the Cyclin-dependent kinase 5 promoter [165].

In addition to epigenetic mechanisms that act on promoter regions, noncoding RNAs also contribute to sex differences in the stress response. MicroRNAs (miRs) are short (~20 nucleotides) non-coding RNAs that act upon mRNAs to suppress protein translation [166]. Male and female mice exposed to variable stress have no overlap in the downregulation of NAc miRs and only 3 overlapping upregulated miRs. Network analysis indicated that many of the genes upregulated by female miRs are immune-related pathways, whereas in males’ upregulation of miRs is associated with neuronal signaling pathways [167]. In male mice exposed to social defeat stress, an miR in peripheral immune cells regulates the behavioral and immune response to stress [168]. Social defeat stress increased the population of immature, pro-inflammatory monocytes (Ly6c^high^) in both susceptible and resilient mice. However, differences in expression of the miR106b~25 cluster in bone marrow-derived bone marrow leukocytes regulated the behavioral response to stress. Leukocyte-specific knockout of this miR cluster promoted resilience. MiR-144-3p in red blood cells has also been identified as a biomarker of depression in humans and stress susceptibility in mice [169]. Furthermore, this miR has the potential to identify who will respond to ketamine treatment or not [169].

Non-coding RNA in sperm can also contribute to epigenetic and transgenerational effects of stress. Paternal stress transmits to the next generation, producing a stress-susceptible phenotype in offspring [170-172]. Both miRs and long non-coding (lncRNA) have been identified as driving these transgenerational effects [170, 172]. LncRNA is implicated in female depression and stress susceptibility. Women with depression have altered primate-specific lncRNAs LINC00473 and Rp11-298d21 (FEDORA) in the PFC [143, 144]. Viral-mediated upregulation of these lncRNAs in the PFC of mice promotes stress resilience or stress susceptibility, respectively, in females but not in male mice. Interestingly, FEDORA expression in the blood was also a potential biomarker of depression for women [143].

**Fig. (2):**
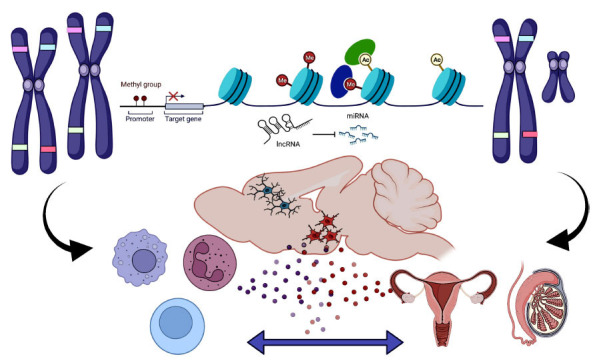
Novel mechanisms of stress susceptibility beyond the HPA axis. Recent rodent studies suggest that multiple epigenetic mechanisms contribute to sex differences in stress susceptibility. These include the ability of alleles on the X chromosome to escape X inactivation in females, DNA methylation and histone modifications that impact the likelihood that a gene will be expressed and noncoding RNAs, including lncRNA and miRs. These mechanisms strongly impact the immune system and gonadal hormones, which also engage in bidirectional communication. Cytokines released by peripheral immune cells and hormones can act in an endocrine fashion to alter immune cells in the brain that, in turn, impact synaptic plasticity. Made with Biorender bly, brexanolone, which is a pharm disorders and depression are intertwined, as experiencing one disease results in an increased risk of developing the other.

Epigenetic regulation of stress/depression also occurs from the ability of immune-associated genes to escape X inactivation (Fig. **2**). The X chromosome contains more immune-related genes than any other chromosome [173], and at least 9 of these genes escape X inactivation, resulting in a larger dose for females [174]. These include the toll-like 7 receptor which is activated by single-strand RNA viruses like COVID-19, and CXCR3, a chemokine receptor downstream of interferon signaling and CD40LG, which modulates T cell communication to B cells. Females have a stronger immune system than males in that they have a more effective response to vaccines, greater production of antibodies, greater release of cytokines during infection, and stronger rejection of tumors and/or transplanted tissue [175]. The tradeoff for this enhanced protection is a greater risk of auto-immune disorders. Women account for 70-80% of the population experiencing autoimmune disorders [174, 175].

Recent research has identified additional peripheral influences that contribute to depression in humans and the stress response in rodents. One theory that is currently being explored is the leaky gut hypothesis [176]. The concept is that stress loosens the intestinal barrier, allowing endotoxins that escape and increase inflammatory signaling in the body and brain. Striking evidence that the gut microbiome contributes to depression symptoms comes from a study that transplanted gut microbes from donors with depression into adult male rats [177]. Rats that got recolonized with microbiota from depressed but not control donors expressed anhedonia and exploratory anxiety-associated behaviors. Ongoing research explores the mechanisms involved and how the gut microbiome can be reshaped to treat mood disorders [178-180]. The gut microbiome can differ by sex [181]. Opposite-sex microbiome transplants confer some of the immune properties of the host [182]; however, more research is needed to understand how sex interacts with gut microbes to shape stress behavioral responses to stress and its relevance to depression.

Young males and females have differences in blood-brain-barrier (BBB) permeability, which can be further altered by stress [183]. Females have greater permeability of the PFC and a more inflamed immune profile at baseline than males [184]. Stress increases the permeability of PFC in females, whereas stress increases the permeability of the striatum in males [185, 186]. These sex differences likely explain why different areas of the brain are more vulnerable to peripheral inflammation in males and females. Increased permeability in response to stress occurs *via* downregulation of the tight junction protein claudin 5. In males, this occurs in the NAc, allowing increased amounts of peripheral cytokines to enter that brain region [185]. In female mice, stress also caused the downregulation of claudin 5 to increase BBB permeability. However, the impact was on the PFC rather than the NAc [186, 187]. The authors also found corresponding changes in genes associated with BBB permeability in post-mortem PFC tissue of women with MDD, suggesting that the PFC may be more vulnerable to neurovascular damage than the NAc in females across species.

Moreover, females have a higher number of reactive microglia, the innate immune cells of the brain within the PFC [188]. Following stress, males express a more reactive immune profile, and females express a greater number of homeostatic markers. Increased microglia activation in the PFC of humans with depression has been identified using post-mortem tissue, and more recently, it was suggested by PET imaging studies that use Translocator protein 4 (TSPO4), which is expressed by microglia, astrocytes and endothelial cells [189]. This initial downregulation of microglia activation in the PFC by females may be a protective compensatory response given the greater permeability of this region. Inversely, microglia from females are activated by stress in the NAc, whereas males seem to be able to suppress activation, maintaining homeostasis [190].

Sex differences in depression and stress responsivity are also influenced by gonadal hormones. Estrogens, progesterone, and androgen receptors are present in most immune cells [191-193]. Because estrogens and progesterone fluctuate across the cycle, they have a dose-dependent impact on immune cell function. Low levels of estrogens stimulate activation of these cells, whereas high levels of estrogens suppress immune function [194]. Studies on stress suggest that estrogen is a modulator of inflammation, as well as a modulator of behavioral responses [151, 195]. Estrogens can increase the secretion of pro-inflammatory interleukins (IL-6 and IL-8) in the innate immune system and increase the secretion of antibodies and regulatory T cells by B cells, increasing the number of regulatory T cells [194]. In general, testosterone suppresses immune activation, particularly by the adaptive immune system [196]. Testosterone induces apoptosis of T cells, resulting in a reduced number in males [197, 198]. Suppressive effects of testosterone on the immune system may, in part, protect males from the immune-mediated effects of stress. For example, testosterone replacement in male or female gonadectomized mice blocked stimulation of the pro-inflammatory cytokine Tumor necrosis factor alpha (TNF-α) with the endotoxin lipopolysaccharide (LPS) [199]. LPS is often used to induce “sickness behavior,” which overlaps with symptoms of depression [200]. In humans, men and women have different immune responses to LPS injection, including higher levels of circulating pro-inflammatory cytokines TNF-α and interleukin-6 along with greater activation of cortisol, whereas men have an increase in the anti-inflammatory cytokine interleukin 10 [201]. Testosterone has a diurnal rhythm, and little is known about how it may differently regulate the immune system during the light *vs*. dark cycle [202]. Most studies that have examined the effect of testosterone on the immune system have examined it in the context of removal or addition. Men who have naturally low levels of testosterone do not exhibit a diurnal rhythm. Further complicating the matter is that cortisol is a regulator of both testosterone secretion and the immune system [202]. In both sexes, the HPA axis traditionally acts to suppress immune responses. As such, sex differences in HPA axis activity also contribute to sex differences in the immune response to stress.

## 6 SEX DIFFERENCES IN DRUG RESPONSE

Depression, anxiety, AD and other stress-related disorders require a multifaced treatment plan, which may include psychotherapy, psychosocial interventions and neuropsychopharmacological treatment, according to the severity of the disorder and the individual patient needs [203]. Several medication classes are available, prominently including selective serotonin reuptake inhibitors (SSRI) and serotonin-noradrenaline reuptake inhibitors (SNRI). Such medications are misleadingly referred to as “antidepressants” [204] but are considered effective treatments for anxiety, obsessive-compulsive disorder and other brain diseases where the monoaminergic neurotransmission may be altered. They are also often prescribed to treat psychiatric symptoms, which are present in neurodegenerative disorders.

To date, there is evidence of sex differences in the pharmacokinetic and pharmacodynamic properties of SSRIs [205-207]. Moreover, it is postulated that many of the pharmacodynamic sex differences may be based on the underlying pharmacokinetic differences between sexes, *i.e*., sex differences in absorption, distribution, metabolism, and excretion. For example, in women, gastric acid secretion is less pronounced, and the gastrointestinal tract transit time is elongated, and as a result, the maximum drug concentration may be reduced [206, 208]. On the other hand, bioavailability is often found to be enhanced in women [209, 210]. Regarding the distribution of drugs, protein binding is less in women, thus increasing the fraction of unbound active drugs [211]. Another important aspect of sex differences in distribution is that women have a larger fat/muscle ratio than men. As CNS-acting drugs must pass the BBB and thus are designed as highly lipophilic, their initial distribution in women is broader, and then they display a lower redistribution rate and clearance. Moreover, hormonal fluctuations during women's menstrual cycle may further affect the absorption and distribution of psychotropics [210, 212]. To date, the most robust evidence regarding pharmacokinetic sex differences is for the metabolism of antidepressants [206, 213]. However, there are no proper guidelines regarding different dosing of most psychotropics in men and women, although generally, women are probably exposed to higher drug levels. In relevance to that, recently, regulatory agencies issued warnings about using lower doses of some hypnotic medications, such as zolpidem, in women [214].

Similar to pharmacokinetics, important sex differences are thought to exist in the pharmacodynamics of many licensed psychotropics [206, 215]. Regarding antidepressants, data is inconclusive and, at times, conflicting. Some studies support the existence of sex differences [205, 216-218], whereas others fail to identify clinically significant differences [219, 220]. As suggested above regarding CRF-antagonists that failed in clinical trials, a possible explanation for these discrepancies is the lack of proper stratification in clinical studies according to sex, as well as to women’s hormonal status [221]. Indeed, when age and hormonal status are considered, premenopausal women respond better to SSRI, whereas older postmenopausal women do not respond as well [217, 222]. The role of estrogens in facilitating drug response was further highlighted by the finding that in post-menopausal women, hormonal replacement therapy co-administered with SSRIs increased favorable outcomes [221, 223]. Several studies produced similar findings, supporting the beneficial interplay between estrogens and antidepressants [224-228]. However, it is worth mentioning that there have been negative studies as well [229, 230], suggesting that the mediating effect of estrogens may be more complicated and context-dependent in various patient populations, according to the underlying neurobiology and especially that of the serotonin transporter (SERT) binding [231].

Pharmacokinetic and pharmacodynamic sex differences are also found in several preclinical studies of psychotropics. Behavioral tests such as the Tail Suspension Test (TST) and the Forced Swim Test (FST) are often used to study the effects of antidepressants. When properly validated, such tests can also highlight important sex differences [232, 233]. For example, compared to males, female rats display higher levels of immobility and lower head shake counts during the FST [45, 58, 234]. Most antidepressants typically reduce immobility and increase swimming duration [223, 235, 236], and female rodents respond more favorably to lower doses of several SSRIs [234, 237-239].

Sex differences may also exist for several other psychotropics, not directly acting on the monoaminergic neurotransmission. Esketamine, a stereoisomer of the racemic drug ketamine, is a newly licensed medication for severe depression and suicidality [240, 241]. Although clinical studies have yet to show important pharmacodynamic sex differences, few preclinical studies suggest sex differences, as females present higher sensitivity to ketamine’s actions than male animals [242]. Moreover, in social isolation stress models, females recovered from depressive-like behaviors with lower doses of ketamine than males [243].

Regarding cholinesterase inhibitors that are mainly used for treating AD, limited data suggest that women respond better to treatment than men [244], but overall, there is an almost complete lack of sex-specific data reported in clinical trials for AD drugs. Also, there is no sex-specific reporting of adverse events related to these treatments [245]. Therefore, more sex-specific designed studies are needed in AD research, as well.

As mentioned, research on therapy for stress-related disorders has focused lately on other targets beyond classical ones. Apart from those already discussed above, these include NMDA receptors and glutamatergic pathways, serotonergic receptors (*e.g*., 5-HT2A as targets of psychedelics), the GABAergic system, neuropeptides, endocannabinoids and many more [246-248]. Another very interesting line of research includes neurosteroids, which are produced *de novo* locally in the brain, as nowadays, it is known that the brain possesses all the enzymes required for the *de novo* synthesis of steroids from cholesterol and not just from steroid precursors synthesized in the gonads or adrenals, which subsequently enter the brain through the bloodstream [249]. Also, there is accumulating evidence that these neurosteroids play a significant role in neuropsychiatric disorders. For example, allopregnanolone, which is the most investigated, is modulated by stress and is involved in PTSD and depression [250]. Notably, brexanolone, which is a pharmaceutical preparation of allopregnanolone, has been licensed as a treatment for post-partum depression [251].

Estrogens can also act as neurosteroids synthesized locally in the brain from steroid precursors, such as testosterone. This conversion is catalyzed by the rate-limiting enzyme aromatase, encoded by the CYP19 gene, and is happening locally in the brain of both males and females [249]. These neuroestrogens are known to be involved in several brain functions, including neuroprotection, cognition and mood [252-254]. As mentioned, testosterone can also derive from *de novo* synthesis locally in the brain or from circulating sources that enter the brain. As known, testosterone is converted to estrogens by aromatase or to non-aromatizable androgens (such as DHT) that have also been found to have an important impact on brain functions, especially cognition and neurodegeneration [249]. As mentioned above, sex steroids, especially testosterone, are known to interact with the HPA axis [255, 256].

Regarding stress studies, there is evidence that the enzyme aromatase is modulated by stress in the hypothalamus of male and female adult quails 257], as well as in the male, but not in the female adult rat hypothalamus 39]. In preclinical models of antidepressant activity, short-term subacute administration of letrozole produced a clear antidepressant effect, which was comparable in effect size to that of fluoxetine, an established antidepressant treatment 258]. However, in other studies of repeated letrozole administration over several days (7-21 days), there were no clear antidepressant behavioral effects despite a persisting modulation of the monoaminergic neurotransmission systems [258, 259]. Sustained aromatase inhibition decreased noradrenaline and dopaminergic activity, as demonstrated by the dopaminergic turnover rates in the hippocampus and PFC of male and female adult rats [39]. Moreover, aromatase inhibition enhanced serotonergic activity, as demonstrated by the serotonin turnover rate in the hippocampus of males and females [39]. These effects were not influenced by adult gonadectomy of rats, which suggests that inhibition of estrogen locally in the brain may play a role [39]. These findings are further supported by the fact that adult ovariectomized aromatase knockout female mice also exhibit enhanced serotonergic activity in the hippocampus, suggesting a modulatory role of neuro-estrogens on hippocampal function [260].

These and other studies suggest that neuro estrogens, as well as their receptors, could be interesting, druggable targets for the treatment of stress-related disorders. Importantly, estrogen’s receptors include classical intracellular ERα and ERβ receptors, as well as the membrane G protein-coupled estrogen receptor 1 (GPER1) receptor, which is involved in rapid, non-genomic actions of estrogens in the brain [261, 262]. ERα and ERβ receptors, as well as their modulators, such as tamoxifen and raloxifene, have long been studied for their role in anxiety, cognition and depression, especially during menopause [249, 263, 264]. ERβ seems to be more involved in mood regulation [264]. Also, tamoxifen has been suggested as a potential treatment for episodes of mania, but more studies are needed [265, 266]. The GPER1 has also been recently involved in stress response and anxiety in male and female mice 267, 268], as well as in depression in men and women [269]. More studies are needed to elucidate the role of pharmacological treatments targeting estrogen receptors in anxiety, affective disorders and AD.

## CONCLUSION

As discussed in detail above, several animal and human studies confirm the existence of various sex differences in the neurobiological mechanisms of stress response that are linked with the pathophysiology of depression, anxiety and AD [138]. However, only recently, preclinical studies started to include both sexes in stress animal models. A significant emphasis has been given by the National Institutes of Health (NIH) policy to consider sex as a biological variable (SABV) in basic research [270]. For practical recommendations on SABV experimental design, the role of gonadal hormones, as well as relevant statistics, the readers are referred to an NIH-funded 18-part video series made by Cohen Veterans Bioscience. These videos are open-access and available online for researchers [271].

Moreover, funding agencies in the USA, Canada, Australia and the European Union vigorously request the inclusion of sex and gender in research, aiming to facilitate better disease understanding [272]. In particular, the inclusion of SABV in neuropsychopharmacology and stress research may significantly increase the translatability of preclinical findings to clinical setups, which in turn can lead to the development of more efficacious treatment for stress-related disorders [273-275]. In fact, the often-observed sex mismatch between preclinical and clinical trials may account for other confounders, for the problem of limited reproducibility in research [276, 277]. Therefore, more sex-aware preclinical research may facilitate the generation of leads for further clinical testing and expedite the early recognition of adverse events that may appear more frequently or at lower doses in one sex or the other [278]. Finally, training a new generation of physicians and health care professionals to account for sex and gender in their practice will pave the way for more personalized care, especially regarding stress-related disorders that, as presented in this review, are heavily characterized by sex-dependent neurobiology.

## LIST OF ABBREVIATIONS

AD: Alzheimer’s Disease
BBB: Blood-brain-barrier
CSF: Cerebrospinal Fluid
FST: Forced Swim Test
GCs: Glucocorticoids
HPA: Hypothalamus-pituitary-adrenal
LC: Locus Coeruleus
LPS: Lipopolysaccharide
MDD: Major Depressive Disorder
miRNA: microRNAs
NAc: Nucleus Accumbens
PTSD: Post-traumatic Stress Disorder
SNRI: Serotonin-noradrenaline Reuptake Inhibitors
TNF-α: Tumor Necrosis Factor Alpha
TST: Tail Suspension Test
